# Collection practices for nontraditional online resources among academic health sciences libraries

**DOI:** 10.5195/jmla.2020.791

**Published:** 2020-04-01

**Authors:** Mary Shultz, Donna R. Berryman

**Affiliations:** Director and Associate Professor, Savitt Medical Library, School of Medicine, University of Nevada, Reno, NV, mshultz@med.unr.edu, http://orcid.org/0000-0002-9236-4375; Head, Health Sciences Library Services, University Libraries, University at Buffalo, Buffalo, NY, donnaber@buffalo.edu, https://orcid.org/0000-0001-8005-4962

## Abstract

**Objective:**

In recent years, individuals and small organizations have developed new online learning and information resources that are often marketed directly to students. In this study, these nontraditional online resources are defined as apps or other online resources that are not available through large and well-known publishers. The purposes of this study are to determine if academic health sciences libraries are licensing nontraditional online resources and to provide a snapshot of current collections practices in this area.

**Methods:**

An online survey was designed and distributed to the email lists of the Collection Development Section of the Medical Library Association and Association of Academic Health Sciences Libraries directors. Follow-up phone interviews were conducted with survey participants who volunteered to be contacted.

**Results:**

Of the 58 survey respondents, 21 (36.2%) reported that their libraries currently licensed at least 1 nontraditional online resource, and 45 (77.6%) reported receiving requests for these types of resources. The resources listed by respondents included 50 unique titles. Of the 37 (63.8%) respondents whose library did not license nontraditional online resources, major barriers that were noted included a lack of Internet protocol (IP) authentication, licenses that charge per user, and affordable institutional pricing.

**Conclusions:**

Evaluation criteria for nontraditional online resources should be developed and refined, and these resources should be examined over time to determine their potential and actual use by students. There is a growing demand for many of these resources among students, but the lack of financial and access models that serve libraries’ needs is an obstacle to institutional licensing.

## INTRODUCTION

Academic health sciences libraries spend significant amounts of money to license online resources to support student learning. Many of these materials are the digital versions of traditional resources such as books, journals, and indexes. Libraries also license point-of-care tools such as UpToDate and DynaMed to support patient care and student learning. Digital formats and an increasing array of multimedia have enhanced libraries’ abilities to meet a variety of learner styles and needs. While these resources differ from those in print-only collections, they are still generally provided by known publishers such as McGraw-Hill, Elsevier, and Wolters-Kluwer.

In recent years, individuals and small organizations that are not traditional publishers have developed new online learning, exam preparation, and information resources. For the purposes of this study, these resources are characterized as “nontraditional online resources” and defined as online resources that have the following characteristics:

- are not produced or provided by traditional publishers
- have no Internet protocol (IP)/proxy authentication available
- are licensed to individual users through individual accounts
- are marketed directly to users and not to libraries

Nontraditional online resources are not a wholly new topic for academic libraries. With the growth of mobile apps, libraries have struggled with criteria for adding apps to collections and websites, considering differences in their licensing models and difficulty in evaluating their quality [1–3]. Some of these same issues regarding evaluation criteria, authentication, and licensing are similar for nontraditional online resources.

O’Hanlon and Laynor have coined the phrase “proprietary study resources” for nontraditional online resources that focus on exam preparation [4]. They define these as “study resources that contain curated content and limit access to individual subscribers.” They describe the challenges that these resources present to libraries’ traditional forms of collection practices, including forms of licenses that do not adhere to common practices and resources that are not well known or vetted. However, these resources continue to grow in popularity and reflect students’ desires for multimedia study options that fit a variety of learning styles.

Requests for nontraditional online resources continue to grow in quantity and volume, especially among medical students. With this growth in use and demand, should libraries support these types of resources? To answer this question, it is necessary to develop a better understanding of the current state of library involvement with nontraditional online resources. The purposes of this study are to determine if academic health sciences libraries are licensing nontraditional online resources and to provide a snapshot of current collection practices in this area.

## METHODS

To determine if and to what extent academic health sciences libraries licensed nontraditional online resources, an online survey was developed (supplemental Appendix A). The first question asked whether the respondents’ libraries licensed nontraditional online resources. Depending on the answer, respondents were directed to different paths of questions through survey logic options. If respondents answered “no” to the first question, additional questions sought to determine the reason. If respondents answered “yes,” additional questions focused on which resources were selected, how and for whom they were licensed, and what issues around funding and costs there were. Informal pretests of the survey were conducted with librarian colleagues at three institutions, and adjustments were made based on their recommendations. Survey respondents were also invited to participate in a follow-up phone interview about nontraditional online resources, based on several guiding questions (supplemental Appendix B). The study was determined to be exempt by the Institution Review Board (IRB) at the University of Nevada, Reno.

In February 2019, an invitation to participate with a link to the survey was sent to two email lists: the Association of Academic Health Sciences Libraries (AAHSL) directors and the Collection Development Section (CDS) of the Medical Library Association (MLA). AAHSL was selected because its members were from libraries that supported medical schools, and a majority of nontraditional online resources supported medical students. CDS was selected because members would have interest in or duties pertaining to collection development in health sciences libraries. At the time of survey distribution, there were 147 members of the CDS list and 167 members of the AAHSL list. The invitation explained the reasons that the survey was being conducted, that participation was voluntary and anonymous, and that the email could be forwarded to other individuals at the recipients’ institutions who were better equipped to answer the questions. The initial invitation was emailed on February 11, 2019, with follow-up reminders on February 21 and March 4. The survey was closed on March 8, 2019.

SurveyMonkey was used to administer the online survey. Results were tallied and analyzed by exporting data to Excel. Survey respondents who volunteered for follow-up phone interviews were contacted and interviewed in May 2019.

## RESULTS

### Survey

Fifty-eight individual complete survey responses were received, with 21 (36.2%) respondents reporting currently licensing at least 1 nontraditional online resource and 37 (63.8%) respondents reporting not currently licensing nontraditional online resources. Of the 58 respondents who completed the survey, 32 (55.1%) were from public institutions, and 26 (44.8%) were from private institutions. Survey results from selected questions are shown in Table 1. Forty-two (72.4%) respondents indicated that their libraries were academic health sciences libraries, 13 (22.4%) were from medical school libraries, 2 (3.4%) were from general academic libraries, and 1 (1.7%) characterized their library as an academic medical center library.

**Table 1:** Survey results

Question	Responses
Total number reporting	58
Licensing nontraditional resources?	
Yes	21
No	37
Licensing for what users?	
For entire institution	11
For defined groups (primarily medical students)	10
Source of funds?	
Materials budget	15
Both materials budget and support from academic unit(s)	4
Student fees	2
Do you have a formal evaluation process for selecting these?	
Yes	12
No	9
Are nontraditional resources covered in your collection policy?	
Yes	3
No	40
Do not have a collections policy	12
Don’t know	3
Have you received requests for nontraditional resources that you have not licensed?	
Yes	45
No	13
Does your institution or a particular unit in your institution (i.e., medical school) require students to directly purchase nontraditional resources?	
Yes	3
No	30
Don’t know	25

Of those who responded regarding their position titles, 30 were library directors, assistant directors, or similar titles, and 14 had position titles that indicated they were part of collection development or content management units. When asked about their primary responsibilities (where respondents could choose more than one area), 41 selected administrative, and 37 selected collection management or development.

Considering libraries that licensed nontraditional online resources (n=21), most respondents became aware of them through suggestions from faculty (n=16) and students (n=12). Fewer respondents learned about them at conferences (n=5), from blogs or other news sources (n=2), or from emails from vendors (n=1). No one received direct mailings or phone calls from vendors. Eleven respondents licensed these resources for their entire institutions, and 12 licensed for subsets of users. Among the latter, medical students were the most frequent subset for whom these resources were licensed (n=8).

**Figure 1:**
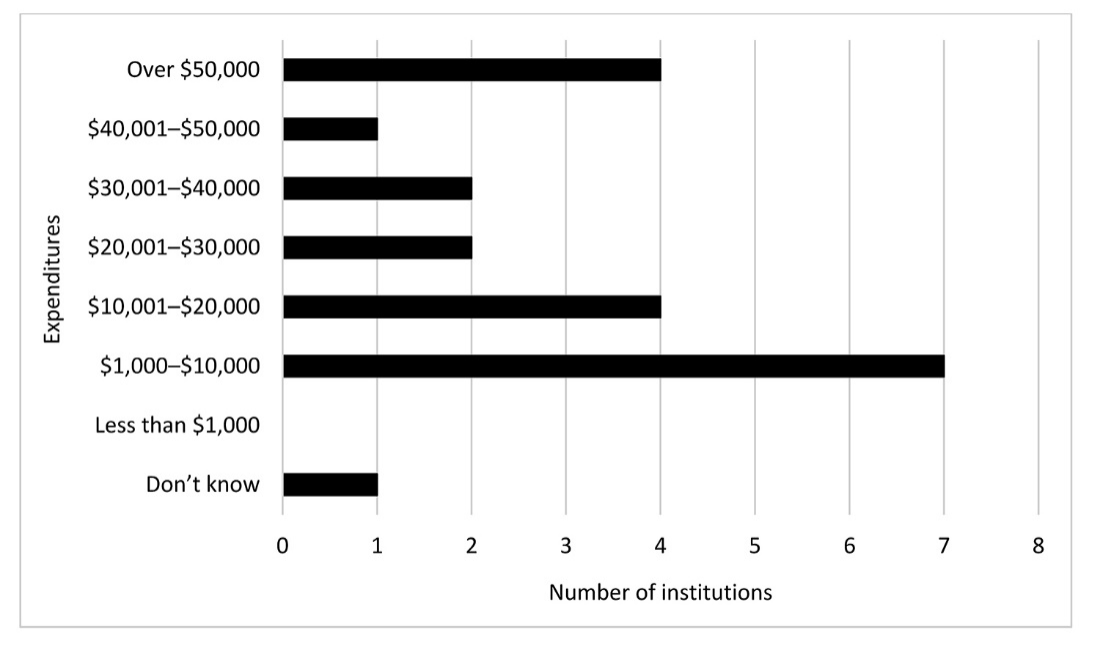
Library expenditures on nontraditional online resources

Figure 1 shows the breakdown of reported spending on nontraditional online resources. Most respondents who licensed nontraditional online resources used their materials budget (n=18), with 3 obtaining funding from academic units and 2 from student fees.

The resources licensed were quite varied (supplemental Appendix C) and included 50 unique resource titles in the combined results of the survey and follow-up interviews. Each resource noted was licensed by only 1 or 2 respondents. Forty-five of the total 58 survey respondents received requests for nontraditional online resources that they were not able to license. Supplemental Appendix D provides a list of 34 unique resource titles from requests received by both survey respondents and interviewees.

Considering respondents whose libraries did not license nontraditional online resources (n=37), the primary reasons indicated (where respondents could choose more than 1 option) were lack of funds (n=20), an expectation that students would purchase access directly (n=16), a prohibition by collection development policy (n=5), and a lack of awareness of these resources (n=8). Only 1 respondent reported offering funding directly to students so that they could purchase their own access to nontraditional online resources.

Licensing resources by individual user and/or for select groups of users posed many challenges, particularly funding. Respondents noted philosophical issues in funding resources for small sectors of their user communities when funding in general was scarce. A sampling of comments received were related to cost:

“Most of the nontraditional resources we are aware of would only benefit a small percentage of our user population, so we can’t justify using our scarce collection funds for these.”

“If funding was not a barrier, we would consider.”

“1. Because funding is very limited, we must continue to prioritize other, traditional resources, that remain core to our collection and mission. Once the essentials are purchased, there is often no money left for anything else. 2. Our current policy is to use library funds to purchase materials that are available to and support all of our schools and departments. As such, spending our limited funds on resources that might be restricted to a certain school or program is not a priority.”

“our budget could not bear the cost per student, and our licensing model is to license for the university at large.”

Many respondents were explicit in opposing licenses that did not offer institution-wide access. A sampling of survey comments showed the contrast between how libraries licensed traditional online resources and the available licensing options from newer vendors:

“Nontraditional resources that have been suggested by students so far have a pricing/access model that is tied to medical students alone. We are only allowed to license resources that are accessible to the entire medical center community and vendors have been unable/unwilling to work with us on coming to agreement on a site license.”

“Across our library system, it’s very rare that we license products that do not offer IP authentication. My understanding is that this helps us avoid confusion (especially when usernames/passwords need to be changed) and it helps make the staff time spent on access inquiries a bit more manageable. IP authentication is also a better way to ensure that access is being appropriately granted to current faculty, students, and staff, whereas handing out an institutional username/password for a resource can get messy.”

“We have decided as a library that we do not have the staff time to give out token/username and password information. We only license content with vendors that allow IP authentication.”

### Interviews

Ten survey respondents volunteered to participate in a follow-up interview, of whom nine licensed nontraditional online resources. Seven were from private institutions, and three were from public institutions. Six described their libraries as academic health sciences libraries, three as medical school libraries, and one as a general academic library.

During the follow-up interviews, interviewees were asked if students actually used nontraditional online resources. Their responses indicated that usage was difficult to determine. One interviewee gave an unqualified “yes” in response, but it was important to note that they were referring to bioinformatics tools, for which vendors generally provided usage data. For many other products, the lack of vendor-supplied data meant that there was no way for librarians to assess product usage.

Two interviewees commented specifically on two resources for which they had vendor-supplied user data: a United States Medical Licensing Exam (USMLE) study prep product and an anatomy study resource. In each case, there was little or no use. One interviewee said:

“We know that if the students were telling us what to buy, these [the USMLE study products] would not be the resources they would buy. We look at the message boards and I talk to students about what they *would* like, and those are items that are licensed on a per-person headcount and we’ve just made a decision not to go down that road.”

During the interviews, it became apparent that there were many different ways that nontraditional online resources were being acquired. Four interviewees said the library itself was the entity handling the licensing of these nontraditional online resources, but they were referring to products that were licensed via traditional site licenses (primarily test prep products). Three interviewees said the library was involved in purchasing products that required individually assigned accounts by providing lists of names and/or emails to the producer of the product, after which library involvement generally ended. One interviewee said that their library developed software that would allow each authorized user to log in and receive an access code to download the product. Two said that at least one individually licensed product was available to students, but the library was not involved in its procurement, which was handled by the medical school. One interviewee described a situation in which the administration of the medical school decided that they would manage the purchasing of individual accounts on behalf of the students for a certain product and said:

“It was a real problem administratively for the School of Medicine to collect everybody’s money and then write a check. Our accounting [department] doesn’t work that way. They did not want that kind of thing going on. So that went away and now the students are pretty much left to their own devices.”

Interviewees were asked what might prompt them to consider licensing nontraditional online resources, and their answers related to funding, demand, and licensing procedures. Four interviewees mentioned additional funding, and four mentioned increased student demand or pressure from administration. One interviewee said:

“If there was agreement at the upper level [that product X or product Y was what the students needed]—but there isn’t. They don’t want to choose a product and we [the library] don’t want to choose a product for them.”

One interviewee remarked that some of these products were highly specialized, saying, “We can’t afford to buy the product for the twelve anesthesiology residents and then there’s a different product—well, they all have specialized products now.” That specialization might add increased cost and present issues related to what another interviewee called “information equity”: providing resources for one group and then being unable to afford specialized products for other groups. Other interviewees remarked that purchasing access per student in the licensing models offered by many providers made the product cost prohibitive. One interviewee said that “new funding streams are needed” to support the licensing of nontraditional online resources.

Echoing the survey results, cost and funding were common themes in the interviews. The primary refrain was that collection budgets were tight, so that adding one new resource often meant that an existing resource needed to be cancelled. However, these nontraditional online resources were, in some ways, an unknown commodity: “We don’t really know what the value proposition is. How useful is it really going to be?” Along the same lines, another interviewee noted that it was difficult to get a good look at some of these products because the lack of institutional access precluded an institutional trial.

Several interviewees mentioned that creating “siloed collections”—resources available only to a portion of the user population—was not in line with their library’s mission or vision. Others talked about the difficulty and staff time required in making a resource available to only a select few:

“When you’re going down the individual user login route, it just becomes so hard to manage and so hard to permission who gets access and who doesn’t. And to get anything like that through our own policies and legal counsel for the medical center, you’ve got to have a good rationale.”

“In terms of administration, if it’s single-user named accounts, if someone leaves the university, do we claim that single-user account back and give it to someone else?”

When asked about the future of these types of resources, interviewees agreed that these products will continue to exist and that the pressure for libraries to find a way to participate in providing access to them will only increase. Many interviewees, however, found some incongruities between libraries’ philosophies and these newer resources:

“It would be against our philosophy if we started building these pockets of collections that only small groups had access to.”

“We always have to keep in mind what the students are demanding. But, really, going down the road of buying—well, basically, you’re buying each student a book if you’re going down the path of buying a block of passwords for a resource. That just really gets away from what libraries have done in the past. We didn’t buy students their own books for their classes. We bought things that could be used as resources for research for everybody. It would change our mission a lot. I would hate to become like a bookstore rather than the library model.”

Finally, some interviewees felt that libraries will have to make some changes or use their long history of managing resources to figure out how to make nontraditional online resources work for libraries as well as users.

“We do a great job of licensing the things that we’ve known for a long time, we’re really on the back end in terms of what users are looking at and using most of the time in their research and their clinical context. I think we’re pretty far behind on what’s really happening.”

“It seems like there are more [nontraditional online resources] coming on the market…that are really integrated with your learning management, your instruction, and there are more layers of integration. So I think the library will have a role there, but I don’t know that the library will be the driver.”

“If we don’t lean into the business [of providing nontraditional online resources], someone else will.”

## DISCUSSION

Most survey respondents (45 out of 58) had received requests for nontraditional online resources that they did not license. Considering this finding, along with the 21 respondents who reported licensing at least 1 nontraditional online resource, the results clearly indicated demand for these types of products. One respondent commented, “If you’ve heard of it, we’ve probably been asked about it”, and another responded, “[we have been asked about] everything. Firecracker, Essential Anatomy, UWorld, Conquest, Combank, Sketchy, Picmonic. The list goes on and on.”

The large variety of resources mentioned by survey respondents and interviewees paired with the small number of existing licenses or requests per resource showed the changing landscape in this area. Resources that appeared to be most highly requested were UWorld, SketchyMedical, and Pathoma.

Many of these products were learning resources that prepared medical students for board exams. According to Warne, “established test prep names like Pathoma, SketchyMedical, First Aid and UWorld fill that demand in a market consisting of more than 40,000 test takers worldwide each year” [5]. Warne went on to surmise that the availability of these products has led to an increase in scores on the USMLE Step 1 Exam. Boards&Beyond provides a lengthy list of student testimonials indicating that use of this product increased their USMLE scores [6]. The perception that these products lead to higher scores continues to drive direct sales to students and demand to libraries.

Most respondents learned of these resources through suggestions from students and faculty rather than through traditional routes. These new vendors generally did not exhibit at library conferences or seek out librarians. It appeared that this new information resource environment was occurring outside what has generally been the information hub: the library. As one respondent commented, “medical libraries get somewhat ousted from the information sphere that our medical trainees are really using.”

While demand was growing, survey respondents noted major barriers to licensing nontraditional online resources, including lack of funding, licenses without institution-wide access, difficulty in evaluating resources, and impact on work flows and collections policies.

Most academic libraries had funding challenges due to the expense of online resources and budgets that were flat or did not keep up with publishers’ yearly increases in pricing. The pricing of nontraditional online resources varied widely. Some online resources that students used were inexpensive, were free, or had free components; others could be quite expensive given that these vendors charged on a per-user basis. For example, in September 2019, the list price for SketchyUltimate was $369.99 per user for a 12-month license [7]. This breaks down to $30.83 per month, which may seem affordable to some students. However, the cost per user may not be affordable for a library that licenses for all university students. Even if licensing for 1 or 2 classes of students, the price can still quickly become unsustainable for many libraries.

Many respondents were quite vocal in opposing licenses that did not offer institution-wide access. This was clear in both the survey and interviews and revealed a major contrast between how libraries licensed traditional online resources and the available licensing options from these newer vendors. Most academic libraries preferred licensing resources via universal IP or proxy access, which allowed authorized users to access resources using a network identification (NetID) and password credentials. Typically for resources licensed per individual, users need to set up their own accounts and remember their chosen user names and passwords. While this is commonplace in today’s online environment, it is an added barrier or step for users, as noted by Boruff and Storie who found that authentication was a barrier to access and recommended more streamlined options for mobile apps [8].

Nontraditional online resources pose challenges to standard collections practices and policies in academic health sciences libraries that extend beyond licensing. Although not specifically related to nontraditional online resources as defined here, direct parallels can be seen in discussions by Arzola and Havelka, DeRosa and Jewell, Saragossi et al., and Boruff and Storie about implications for collections when considering library provision of mobile apps [1–3, 8]. DeRosa and Jewel describe decision criteria including quality of content, reputation of the publisher (not easily determined for apps or nontraditional online resources), cost, licensing options, copyright, and fair use issues [2]. Saragossi et al. suggest consideration of similar issues along with the availability of COUNTER-compliant statistics [3]. Accessibility compliance should be added to these considerations.

Saragossi et al. also note that libraries that provide nontraditional online resources often manage individual user accounts, a tedious process that can have a serious impact on library work flows [3]. With individual accounts, it is inevitable that some users will forget their user names and/or passwords and contact library staff for assistance. In these cases, library staff must know which users have access to which resources as well as how to troubleshoot each product, which can impact service to library users. Alternatively, as Boruff and Storie suggest, users can seek help from friends or colleagues and not recognize the library as a source of technical support [8].

Another consideration is the recognition of libraries’ contribution: if libraries license resources on an individual basis, requiring users to log in with their personal accounts rather than through the library’s system removes a significant reminder that the resource is provided by the library. If a resource is downloaded onto a computer, tablet, or mobile device, users may entirely forget that access has been made possible by the library.

Adding these resources to libraries’ collections may also require revisions to evaluation practices. O’Hanlon and Laynor recommend working with faculty and students to develop evaluation criteria for these newer resources [4]. As many of these resources are designed for particular groups of users (e.g., medical students, research faculty), representatives from these groups may need to be recruited to serve on evaluation teams prior to licensing a resource. This may also require trial access periods, which may be unfamiliar territory for newer vendors.

Many vendors do not currently seem willing to consider other models of licensing and pricing that would appeal to institutions. This behavior could simply reflect the point at which vendors are in their corporate development. Some resources initially licensed directly to individual users and later moved to institutional licensing models. The most notable is UpToDate, which focused on individual licensing in its early years. Hurdles in the move to a broader, larger audience may be too much for some start-up companies. It is also possible that many resources will fade away and be replaced by others. If demand for these resources continues, companies may consider licensing models that are more beneficial to libraries.

### Limitations

The survey response rate could not be calculated with accuracy due to the use of electronic mailing lists as a mode of recruitment. For instance, some individuals likely belonged to both the AAHSL directors and MLA CDS lists but would have only completed one survey. Also, the recruitment email suggested that the survey invitation could be forwarded to the most appropriate party at an institution who could respond. Completion of the survey was voluntary, and responses by institution were not tracked.

Examples of nontraditional online resources were defined and described in the recruitment emails and on the initial page of the survey (supplemental Appendix A). However, some responses indicated confusion about what constituted a nontraditional online resource. For example, one respondent listed AnatomyTV as a resource in this category, and at least one respondent listed several genomic information resources, which would all be considered traditional resources per the provided definition. O’Hanlon and Laynor recently proposed the name “proprietary study resources” for online resources intended for exam preparation that are licensed to individuals [4]. The present study encompassed these resources as well as online resources that were not specific to exam preparation by considering the broader category of “nontraditional online resources.”

## CONCLUSIONS

The findings of this study indicate a strong and growing demand for nontraditional online resources. Licensing the types of resources may create truly demand-driven collections where users’ expectations are for an individual, customized collection that changes over time to meet their needs at the time of need, resulting in a “precision medicine” view of library collections. Is this growing demand for nontraditional online resources an early indicator of change in the philosophy of collection development? O’Hanlon and Laynor suggest that health sciences librarians “reevaluate some traditional trains of thought” about these types of resources [4]. Perhaps it is time to consider customized models of collection development and management that have been eschewed in the past in favor of broader access. On the other hand, it is possible that vendors may change their licensing models. If vendors of nontraditional online resources were to move to true institutional licensing with IP authentication, more users and students would be reached, and their market share as well as user loyalty could grow.

## SUPPLEMENTAL FILES

Appendix A Licensing of nontraditional resources by academic health sciences libraries survey questions

Appendix B Guiding questions for follow-up phone interviews

Appendix C Nontraditional resources that respondents license (survey and interview results)

Appendix D Nontraditional resource requests (survey and interview results)

## 

**Mary Shultz, MSLIS**, mshultz@med.unr.edu, http://orcid.org/0000-0002-9236-4375, Director and Associate Professor, Savitt Medical Library, School of Medicine, University of Nevada, Reno, NV

**Donna R. Berryman, MLIS, EdD, AHIP**, donnaber@buffalo.edu, https://orcid.org/0000-0001-8005-4962, Head, Health Sciences Library Services, University Libraries, University at Buffalo, Buffalo, NY
