# Supplementary material for: Collection practices for nontraditional online resources among academic health sciences libraries
Source: J Med Libr Assoc. 2020 Apr 1;108(2):253–61. doi: 10.5195/jmla.2020.791 (PMC7069827; doi:10.5195/jmla.2020.791)
Supplement: Appendix A [file jmla-108-253-s001.pdf]

Journal of the Medical Library Association

Supplemental content to
J Med Libr Assoc. Apr;108(2):dx.doi.org/10.5195/jmla.2020.791
www.jmla.mlanet.org
© Shultz, Berryman 2020

Collection practices for nontraditional online resources among academic health sciences libraries

Mary Shultz, MSLIS; Donna R. Berryman, MLIS, EdD, AHIP

APPENDIX A
Licensing of nontraditional resources by academic health sciences libraries survey questions

This survey pertains to nontraditional online health sciences resources. We define these as apps or other online resources that are not available through large and well-known publishers such as Wiley and Elsevier. Nontraditional resources are those that are directly licensed from the individuals or small organizations that produce them. These resources typically do not provide licensed Internet protocol (IP) access but instead provide individual accounts, and students often purchase or license them directly.

For example, SketchyMedical is a nontraditional resource. It is a visual learning system that provides graphical representations of key concepts in basic sciences. This is produced and licensed by SketchyMedical. Generally, students purchase access directly from SketchyMedical. This vendor does not offer IP access but does provide bulk pricing for institutions who license on behalf of students. There are many other products that license in a similar fashion, including Pathoma and UWorld.

1. Do you license any nontraditional resources?
- ☐ Yes *[Logic moves to question 2]*
- ☐ No *[Logic moves to question 9]*

2. Which resources do you license? Check all that apply.
- ☐ SketchyMedical
- ☐ Pathoma
- ☐ UWorld
- ☐ Complete Anatomy
- ☐ Essential Anatomy
- ☐ Anki
- ☐ Notability
- ☐ iAnnotate
- ☐ GoodReader
- ☐ Heart Murmurs Pro
- ☐ Other, please list below *[includes text box]*

3. How did you find out about the resource(s)? Check all that apply.
- ☐ Suggestion from students
- ☐ Suggestion from faculty
- ☐ Conferences
- ☐ Email from vendor
- ☐ Phone call from vendor
- ☐ Direct mail
- ☐ Blogs or other news sources
- ☐ Other, please describe below *[includes text box]*

JMLA Journal of the Medical Library Association

Supplemental content to
J Med Libr Assoc. Apr;108(2):dx.doi.org/10.5195/jmla.2020.791
www.jmla.mlanet.org
© Shultz, Berryman 2020

4. Do you have a formal evaluation process when selecting these types of resources?
- ☐ Yes
- ☐ No

5. Do you license these resources for all of your institution's users as opposed to a specific subset (i.e., medical students only)?
- ☐ Yes, all university users *[Logic moves to question 7]*
- ☐ No, license for subsets of users *[Logic moves to question 6]*

6. For which subsets of users do you license nontraditional resources? Check all options that apply.
- ☐ Health sciences students
- ☐ Medical students
- ☐ Nursing students
- ☐ Dentistry students
- ☐ Pharmacy students
- ☐ Veterinary students
- ☐ Other, please list below *[includes text box]*

7. Approximately how much does your library spend each year on these types of resources?
- ☐ Less than $1,000
- ☐ $1,001–$10,000
- ☐ $10,001–$20,000
- ☐ $20,001–$30,000
- ☐ $30,001–$40,000
- ☐ $40,001–$50,000
- ☐ Over $50,000
- ☐ I don't know

8. What are your major sources of funding for these types of resources? Check all that apply.
- ☐ Materials budget
- ☐ Donor support
- ☐ Special monetary support from academic units
- ☐ Student fees
- ☐ I don't know
- ☐ Other sources of funding, please describe below *[includes text box]*

9. If you do not license nontraditional resources, please tell us why not? Check all that apply.
- ☐ No available funding
- ☐ Unaware of these resources
- ☐ Expect students to purchase directly
- ☐ Not permitted per collection development policy
- ☐ Other, please explain below *[includes text box]*

10. Alternatively, do you offer funding directly to students so that they can purchase their own apps/online resources?
- ☐ Yes *[Logic moves to question 11]*
- ☐ No *[Logic moves to question 12]*

JMLA Journal of the Medical Library Association

SC
Supplemental content to
J Med Libr Assoc. Apr;108(2):dx.doi.org/10.5195/jmla.2020.791
www.jmla.mlanet.org
© Shultz, Berryman 2020

11. Since you indicated that you have a fund that pays money directly to students for purchasing these resources, can you provide us with more information? For example, the size of the fund, the application process, the decision criteria, the eligibility criteria?
[Text box provided for answer]

12. Does your institution or a particular unit in your institution (i.e., medical school) require students to directly purchase nontraditional resources?
- ☐ Yes
- ☐ No
- ☐ I don't know

13. Have you received requests for nontraditional resources that you have not licensed?
- ☐ Yes
- ☐ No
- ☐ If you selected Yes, please list *[includes text box]*

14. Are nontraditional resources addressed in your collections policy?
- ☐ Yes
- ☐ No
- ☐ I don't know
- ☐ My library does not have a collection development policy

If you selected Yes, please describe *[includes text box]*

15. Are there any other comments you would like to make about nontraditional resources?
[Text box provided for answer]

16. What type of institution do you work in?
- ☐ Public
- ☐ Private

17. How would you describe your library?
- ☐ Academic health sciences library
- ☐ Medical school library
- ☐ Hospital library
- ☐ Other
- ☐ If you selected Other, please describe *[includes text box]*

18. What is your job title?
[Text box provided for answer]

19. What are your primary responsibilities? Check all that apply.
- ☐ Administrative
- ☐ Collection management/development
- ☐ Reference
- ☐ Liaison librarian
- ☐ Other

If you selected Other, please describe *[includes text box]*

JMLA Journal of the Medical Library Association

SC
Supplemental content to
J Med Libr Assoc. Apr;108(2):dx.doi.org/10.5195/jmla.2020.791
www.jmla.mlanet.org
© Shultz, Berryman 2020

20. Thank you for taking our survey. We really appreciate it! If you are willing to be interviewed by phone about this, please provide us with your contact information. This will be kept confidential.

If you don't wish to be interviewed, simply select the Finish button at the bottom.
Name
Institution
City/Town
State/Province
Email address
Phone number
[Text boxes provided for answers]
