# Supplementary material for: Collection practices for nontraditional online resources among academic health sciences libraries
Source: J Med Libr Assoc. 2020 Apr 1;108(2):253–61. doi: 10.5195/jmla.2020.791 (PMC7069827; doi:10.5195/jmla.2020.791)
Supplement: Appendix B [file jmla-108-253-s002.pdf]

JMLA Journal of the Medical Library Association

SC
Supplemental content to
J Med Libr Assoc. Apr;108(2):dx.doi.org/10.5195/jmla.2020.791
www.jmla.mlanet.org
© Shultz, Berryman 2020

Collection practices for nontraditional online resources among academic health sciences libraries

Mary Shultz, MSLIS; Donna R. Berryman, MLIS, EdD, AHIP

APPENDIX B

Guiding questions for follow-up phone interviews

1. Your contact information was not tied to your responses, so could you tell us about your experience with nontraditional online resources? Have you licensed any?
    a. If no, why not? *[Then proceed to question #8]*
    b. If yes, which ones?
2. When and for how long did you license these?
3. How did you hear about these resources?
4. What led you to consider these types of resources?
5. Do you feel they are getting used by students? By faculty? Have the vendors provided you with usage stats?
6. Will you renew or have your renewed them?
7. What could have gone better? Was there anything you did not like about the products or licensing process?
8. Do you know if students are licensing products directly? If so, do you know about how much they pay annually?
9. What, if anything, might prompt you to consider licensing these types of resources?
10. How do you think libraries will be thinking about or dealing with these types of resources in, say, five years?

Do you have any other comments you would like to make?

Do you have any questions for us?
