# Supplementary material for: Collection practices for nontraditional online resources among academic health sciences libraries
Source: J Med Libr Assoc. 2020 Apr 1;108(2):253–61. doi: 10.5195/jmla.2020.791 (PMC7069827; doi:10.5195/jmla.2020.791)
Supplement: Appendix C [file jmla-108-253-s003.pdf]

JMLA Journal of the Medical Library Association

SC

Supplemental content to
J Med Libr Assoc. Apr;108(2):dx.doi.org/10.5195/jmla.2020.791
www.jmla.mlanet.org
© Shultz, Berryman 2020

Collection practices for nontraditional online resources among academic health sciences libraries

Mary Shultz, MSLIS; Donna R. Berryman, MLIS, EdD, AHIP

APPENDIX C

Nontraditional resources that respondents license (survey and interview results)

Which resources do you license?	Uniform resource locator (URL)	Number of responses	Description of resource
3D4medical	https://3d4medical.com/	1	3D anatomy resources.
American College of Radiology (ACR) Continuous Professional Improvement	https://www.acr.org/Lifelong-Learning-and-CME/Learning-Activities/CPI	1	Continuing medical education (CME) in essential areas of ACR-required study.
American Hospital Association (AHA) data products	https://www.aha.org/data-insights/aha-data-products	1	AHA offers various data products including their annual survey data set and data viewer that allows users to create custom data files from the database.
Amirsys Imaging Reference Center	https://www.elsevier.com/about/press-releases/clinical-solutions/elsevier-acquires-amirsys,-provider-of-healthcare-information-solutions-and-content-for-radiology,-pathology,-and-anatomy	1	Diagnostic imaging resource acquired by Elsevier.
AnatomyTV	https://www.anatomy.tv/	1	3D anatomy resource.

Supplemental content to
J Med Libr Assoc. Apr;108(2):dx.doi.org/10.5195/jmla.2020.791
www.jmla.mlanet.org
© Shultz, Berryman 2020

Which resources do you license?	Uniform resource locator (URL)	Number of responses	Description of resource
Aquifer (formerly, MedU)	https://www.aquifer.org/	3	Provides online courses, patient cases, exams, and assessment for medical students and faculty.
BoardVitals	https://www.boardvitals.com/	2	Preparation for board exams and certification for a variety of specialties.
BrowZine	https://browzine.com	1	Current awareness tool that allows users to view tables of content and articles on various devices.
Canopy Medical Spanish	https://withcanopy.com/	1	Online resource for learning medical Spanish.
ChemOffice	http://www.cambridgesoft.com/Ensemble_for_Chemistry/details/Default.aspx?fid=16	1	Productivity software providing applications to track scientific work, draw molecules and reactions, link to electronic lab notebooks, create 3D models, etc.
Child Abuse Atlas from Evidentia Publishing	https://www.childabuseatlas.com	1	Educational platform to build skills to evaluate and diagnose child (sexual) abuse. Includes e-learning modules and case review.
CLC Genomics Workbench (Biomedical Genomics Workbench)	https://www.qiagenbioinformatics.com/products/clc-genomics-workbench/	1	From Qiagen, data analysis tools.
COMQUEST	https://comquestmed.com/	1	Test prep resource for osteopathic exams: Comprehensive Osteopathic Medical Licensing Examination (COMLEX-USA) and Comprehensive Osteopathic Medical Achievement Test (COMAT).
Covidence	https://www.covidence.org/home	2	Systematic review management resource.

JMLA Journal of the Medical Library Association

Supplemental content to
J Med Libr Assoc. Apr;108(2):dx.doi.org/10.5195/jmla.2020.791
www.jmla.mlanet.org
© Shultz, Berryman 2020

Which resources do you license?	Uniform resource locator (URL)	Number of responses	Description of resource
DistillerSR	https://www.evidencepartners.com/products/distillersr-systematic-review-software/	1	Systematic review management resource.
Doc.Com	https://doc.com/	1	Provides medical information from doctors to patients.
Dollars for Docs (ProPublica)	https://projects.propublica.org/docdollars/	2	Tracks payments from pharmaceutical and medical device companies to physicians.
Electronic Nutrition Care Process Terminology (eNCPT)	https://www.ncpro.org/	1	A standardized terminology to ensure optimal nutrition care.
ePath3D	http://www.epath3d.com/	1	Software that enables users to draw pathways and biological graphics.
Exam Master	https://exammaster.com/	2	Online test prep resource for United States Medical Licensing Exam (USMLE), National Council Licensure Examination (NCLEX), Physician Assistant National Certifying Exam (PANCE), etc.
ExpertPath	https://www.expertpath.com/	1	Decision support tool for clinical pathology.
FAIR Health Consumer	https://www.fairhealthconsumer.org/	1	Provides information and cost estimates for medical and dental procedures.
GeneSpring GX	https://www.agilent.com/en/products/software-informatics/life-sciences-informatics/genespring-gx	1	Statistical tools for data analysis and visualization.

Supplemental content to
J Med Libr Assoc. Apr;108(2):dx.doi.org/10.5195/jmla.2020.791
www.jmla.mlanet.org
© Shultz, Berryman 2020

Which resources do you license?	Uniform resource locator (URL)	Number of responses	Description of resource
Grammarly	https://www.grammarly.com	1	Online resource to improve grammar.
Human Gene Mutation Database (HGMD)	https://digitalinsights.qiagen.com/products-overview/clinical-insights-portfolio/human-gene-mutation-database/	1	Human gene mutation database from Qiagen.
Immunoquery	https://www.immunoquery.com/	1	An evidence-based decision support system for immunohistochemistry.
Ingenuity Pathway Analysis from Qiagen	https://www.qiagenbioinformatics.com/products/ingenuity-pathway-analysis/	2	From Qiagen, data analysis tool for interpreting experimental results.
Ingenuity Variant Analysis	https://www.qiagenbioinformatics.com/products/ingenuity-variant-analysis/	2	From Qiagen, tool that analyzes findings to find causal variants.
iThenticate	http://www.ithenticate.com/	1	Plagiarism detecting software.
LinkedInLearning (formerly, lynda.com)	https://www.linkedin.com/learning/	1	Online learning modules.
MetaCore, Cortellis	https://clarivate.com/products/metacore/	1	Pathway analysis and knowledge mining tool from Clarivate.
NetAnatomy	http://www.netanatomy.com/	1	Radiographic, cross-sectional, and gross anatomy resource.

JMLA Journal of the Medical Library Association

Supplemental content to
J Med Libr Assoc. Apr;108(2):dx.doi.org/10.5195/jmla.2020.791
www.jmla.mlanet.org
© Shultz, Berryman 2020

Which resources do you license?	Uniform resource locator (URL)	Number of responses	Description of resource
Notability	https://apps.apple.com/us/app/notability/id360593530	1	Note taking and sketching tools.
Nutrition Care Manual (NCM)	https://www.nutritioncaremanual.org/	1	Online resource providing evidence-based nutrition information, client education library, nutrition recommendations, and customizable diet manual.
OnlineMedEd	https://onlinemeded.org/	1	Resource for clinical learning and prep for shelf and board exams.
Partek Flow	http://www.partek.com/partek-flow/	1	Analysis software for sequencing data applications.
Partek Genomics Suite	http://www.partek.com/partek-genomics-suite/	1	Suite of products for statistical analysis, work flows, visualizations, etc.
Pathoma	https://www.pathoma.com/	3	Pathology resource with video lectures and more.
Physician Assistant Exam Prep	https://apps.apple.com/us/app/physician-assistant-exam-prep/id1126045571	1	Preparation for the Physician Assistant National Certifying Exam.
PolicyMap	https://www.policymap.com/	1	Data and mapping tool.
QxMD	https://qxmd.com/	1	Current awareness services (READ), calculators, and mobile learning platform.
ScienceSlides Online	http://www.scienceslides.com/	1	Assists lecturers in preparing slides in biomedical fields by providing objects, templates, etc.

JMLA Journal of the Medical Library Association

Supplemental content to
J Med Libr Assoc. Apr;108(2):dx.doi.org/10.5195/jmla.2020.791
www.jmla.mlanet.org
© Shultz, Berryman 2020

Which resources do you license?	Uniform resource locator (URL)	Number of responses	Description of resource
SketchyMedical	https://sketchymedical.com/	1	Graphical learning tool for microbiology, pathology, pharmacology, and USMLE prep.
SnapGene	https://www.snapgene.com/	1	Data software to analyze, visualize, and document research procedures. Provides visualization and simulation for DNA manipulations.
STAT + from STAT News	https://www.statnews.com/stat-plus/	1	Provides biotech, pharmaceutical, policy, and life science news coverage and analysis.
STATPlus for Windows	https://www.analystsoft.com/en/products/statplus/	1	Suite of statistics tools and graphical analysis methods.
UpToDate ("handling in a nontraditional way")	https://www.uptodate.com/home	1	Point-of-care tool for clinical decision making.
USMLE Easy	https://www.usmle-easy.com/	2	Exam prep for USMLE.
UWorld (USMLE World)	https://www.uworld.com/	2	Exam prep resource for USMLE, Medical College Admission Test (MCAT), NCLEX, and more.
Visible Body	https://www.visiblebody.com/	1	Anatomy learning tool.
