# Supplementary material for: Collection practices for nontraditional online resources among academic health sciences libraries
Source: J Med Libr Assoc. 2020 Apr 1;108(2):253–61. doi: 10.5195/jmla.2020.791 (PMC7069827; doi:10.5195/jmla.2020.791)
Supplement: Appendix D [file jmla-108-253-s004.pdf]

JMLA Journal of the Medical Library Association

Supplemental content to
J Med Libr Assoc. Apr;108(2):dx.doi.org/10.5195/jmla.2020.791
www.jmla.mlanet.org
© Shultz, Berryman 2020

Collection practices for nontraditional online resources among academic health sciences libraries

Mary Shultz, MSLIS; Donna R. Berryman, MLIS, EdD, AHIP

APPENDIX D

Nontraditional resource requests (survey and interview results)

Requested resources not licensed	Uniform resource locator (URL)	Number of responses	Description
3D4medical	https://3d4medical.com/	1	3D anatomy resources.
Advisory Board	https://www.advisory.com/about-us	1	Provides health care executives research and information for developing strategies, increasing performance improvement and team effectiveness.
AMBOSS	https://www.amboss.com/us	2	A learning resource with question bank, learning cards, custom sessions by organ or symptom.
Aquifer (formerly, MedU)	https://www.aquifer.org/	1	Provides online courses, patient cases, exams, and assessment for medical students and faculty.
BioRender	https://biorender.com/	1	Tool to create professional scientific figures.
Biosphera3D	https://biosphera3d.com	1	3D tool for veterinary anatomy.
Boards&Beyond	https://www.boardsbeyond.com/	1	A board review system for United States Medical Licensing Exam (USMLE) Step 1 featuring over 400 videos and quiz questions.

JMLA Journal of the Medical Library Association

Supplemental content to
J Med Libr Assoc. Apr;108(2):dx.doi.org/10.5195/jmla.2020.791
www.jmla.mlanet.org
© Shultz, Berryman 2020

Requested resources not licensed	Uniform resource locator (URL)	Number of responses	Description
BoardVitals	https://www.boardvitals.com/	2	Preparation for board exams and certification for a variety of specialties.
Canopy Medical Spanish	https://withcanopy.com/	1	Online resource for learning medical Spanish.
Clinical Constellation	https://www.skyscape.com/product/Clinical-Constellation-All-in-One-Clinical-Solution	1	A resource that bundles several books in a mobile format to assist with medical decision making for primary care.
Combank by TrueLearn	https://truelearn.com/comlex-usa/	1	Exam review and preparation for osteopathic medicine Comprehensive Osteopathic Medical Licensing Examination (COMLEX-USA) exams.
COMQUEST	https://comquestmed.com/	1	Test prep resource for osteopathic exams: COMLEX-USA and Comprehensive Osteopathic Medical Achievement Test (COMAT).
Cortellis Drug Discovery Intelligence (formerly, Integrity from Clarivate Analytics)	https://clarivate.com/cortellis/campaigns/introducing-cortellis-drug-discovery-intelligence/	1	Pharmaceutical and drug development intelligence.
Draw-it-to-Know-it	https://drawittoknowit.com/	1	Graphical system allowing students to watch and listen to videos with illustrations of concepts, then redraw those illustrations.
ExpertPath	https://www.expertpath.com/	2	Decision support tool for clinical pathology.
face2gene	https://www.face2gene.com/	1	Suite of phenotyping applications that facilitate genetic evaluations.

Supplemental content to
J Med Libr Assoc. Apr;108(2):dx.doi.org/10.5195/jmla.2020.791
www.jmla.mlanet.org
© Shultz, Berryman 2020

Requested resources not licensed	Uniform resource locator (URL)	Number of responses	Description
Firecracker	http://firecracker.lww.com/	4	Learning system for medical and physician assistant students to prepare for USMLE and Physician Assistant National Certifying Exam (PANCE).
Healthcare Cost and Utilization Project (HCUP)	https://www.hcup-us.ahrq.gov/	1	HCUP collection of longitudinal hospital care data in the United States.
Kaplan	https://www.kaptest.com/usmle	4	Test preparation for USMLE.
Lecturio	https://www.lecturio.com	1	Online "tutor" system for exam preparation, includes videos and question banks.
Medical Knowledge Self-Assessment Program (MKSAP)	https://mksap.acponline.org/	1	Learning resource and board prep for internal medicine physician and residents.
MetaDrug from Clarivate Analytics	https://portal.genego.com/	1	Part of the MetaCore product. Includes manually curated information on the effects of small molecule compounds.
Netter's Anatomy Atlas 7e	https://apps.apple.com/us/app/netters-anatomy-atlas-7e/id1356687853	1	Anatomy Atlas app.
Oncomine Assays for Oncology Research from Thermo Fisher Scientific	https://www.thermofisher.com/us/en/home/clinical/preclinical-companion-diagnostic-development/oncomine-oncology.html	1	Provider of assays for oncology diagnostic development.

JMLA Journal of the Medical Library Association

SC
Supplemental content to
J Med Libr Assoc. Apr;108(2):dx.doi.org/10.5195/jmla.2020.791
www.jmla.mlanet.org
© Shultz, Berryman 2020

Requested resources not licensed	Uniform resource locator (URL)	Number of responses	Description
OnlineMedEd	https://onlinemeded.org/basic-sciences	2	Learning system that includes online lectures/videos, flashcards, questions, and coaching.
Osmosis	https://www.osmosis.org/	3	Learning platform with study tools and materials including videos, questions, and flashcards.
Pathoma	https://www.pathoma.com/	6	Pathology resource with video lectures and more.
Physeo	https://physeo.com/	1	USMLE Step 1 review resource that includes online text, videos, and practice questions.
picmonic	https://www.picmonic.com/	1	Picture mnemonics for study aids.
QxMD-READ	https://qxmd.com/read-by-qxmd	1	Current awareness services (READ), calculators, and mobile learning platform.
Rosetta Stone	https://www.rosettastone.com/	1	Language learning system.
SketchyMedical	https://sketchymedical.com/	11	Graphical learning tool for microbiology, pathology, pharmacology, and USMLE prep.
USMLE Rx	https://www.usmle-rx.com/	1	Exam prep resource for USMLE. Includes questions, videos, and flashcards.
Uworld (USMLE World)	https://www.uworld.com/	16	Exam prep resource for USMLE, Medical College Admission Test (MCAT), NCLEX, and more.
